# Clinical Cases

**Published:** 1889-05

**Authors:** E. W. Berridge

**Affiliations:** London


### CLINICAL CASES.

E. W. BERRIDGE, M. D., LONDON.

(15) *Sepia.*—August 27th, 1885.—Mr. B. wrote that he had been ailing for some time; aching in calves, extending up to knees, with a feeling almost as if the bones were decaying. The excitement and occupation of business during the morning makes him forget his aching limbs. Has had much worry lately. *Sepia*$^{cm}$ (F. C.) twice daily for eight days soon cured.

(16) *Lac felinum.*—Mr. E., aged twenty-eight, March 26th, 1888, consulted me for iritis. I first prescribed for him August 18th, 1884, when he told me that he had had six attacks of inflammation of the eyes, alternating in either eye in the three previous years. It had been diagnosed as iritis at an ophthalmic hospital. The attack would last four or five weeks. The last two attacks had been five months apart; generally there was an interval of six months; never more than eight. He never had syphilis. I gave him according to the changing symptoms *Acon.*, *Natr-mur.*, and *Zinc.* He was well by September 15th, but had improved much sooner than usual; the attack having lasted nine days when I first saw him. Two allopaths had told him he never would be cured.

On January 9th, 1885, he had another attack in right eye, which I cured with *Zinc*, followed by *Merc.* He was nearly well by January 31st, and said it was the shortest attack he had ever had.

On January 2d, 1886, had another attack in right eye, having remained well longer than ever before. I cured it with *Acon.* and *Merc.*

He remained quite well till March 17th, 1888 (more than two years), then the right eye was attacked during the excessively cold weather. I saw him March 26th. Right eye was much inflamed ; aching in it at night; *shooting from right eye to temple* and eyebrow, worse at night between three and five A. M. Has been worse since 22d. Sight very hazy. Iris looks dull. Since 17th a black spot before right eye, moving with the eye, when in sunlight. *Lac felinum*$^{cm}$ (Fincke) every four hours.

April 4th.—Took the last dose last night; after the third day took it only thrice daily. He improved the first night, the improvement increasing each night; the third night the pain did not wake him at all. No return of the aggravation between three and five A. M. since commencing the medicine. The shooting pain, which was the last to appear, was the first to disappear. Sight not nearly so hazy; can see a red stone in a ring which he could not before. Eye looks clearer. Yesterday morning, feeling of grit in right eye on waking, lasting two or three hours (? effect of medicine). No more medicine given.

April 19th.—Has felt quite well for five days, and has gone back to his work, which is wheeling a bath chair. No black speck. Sight getting clearer daily ; can read print now. Eye looks natural, and there has been no pain in it for eight or ten days. Since April 2d, and worse for the last two weeks, inner side of arch of right foot is swelled, with aching pain, worse at night if he wakes and moves, and he has to limp on first getting out of bed; the pain ceases after walking about ten minutes, but if he rests he feels it when he moves again ; the pain is as if the muscles there were contracted.

These symptoms were like those of *Rhus*, but as the transference of symptoms from the eye to the foot was a curative effect, I gave no medicine. Invariably when symptoms pass from above downward, or from within outward, or from a more important to a less important organ, the medicine should be allowed to act without repetition or interference. It is only when the new symptoms persist that further medicine should be given. I

once greatly relieved a case of chronic headache with *Nux;* her feet became more painful, and to relieve them I prescribed *Kali.* The feet improved; but the headache returned as badly as ever, and the patient gave up treatment. I know better now.

June 13th.—No return of pain in eye, but black spot has returned. No swelling of foot or pain at night. Foot feels sore on first standing, and stiff after sitting, but not nearly so bad as before.

I have not seen him since; but as he was a free patient and had never been relieved before he tried Homœopathy, I conclude he is still well.

(17) *Sulphur.*—April 18th, 1885.—Miss A. says she caught cold in right eye about two months ago, which caused lachrymation and pricking pains. This she cured (?) by application of rose-water. About three weeks ago both eyes became affected, and this time rose-water did no good. She has now constant lachrymation, of a rather gelatinous character, all day, but worse morning and evening. In the morning sees a halo round artificial light, yellow inside, then green, then yellow outside; removed by washing. Gaslight looks dim. Smarting in external canthi, which are red and prick at times. *Sulphur*$^{dm}$ (F. C.) once daily for seven days.

April 26th.—Writes that eyes are really better; symptoms continue, but are all less marked, and eyes do not pain nearly so much; the halo is thinner and smaller, and sight less dim in evening. No medicine.

May 11th.—Writes that she is almost well; eyes are only a little weak; a pricking sensation in them when exposed to wind; lids very heavy on first waking in morning. Halo quite gone for more than a week.

June 24th.—Reports she has remained quite well.

(18) *Natrum muriat.*$^{cm}$ (F. C.)—Removed an inability to blow the nose, the mucus getting into throat.

(19) *Rhus tox.*—May 27th, 1885.—Miss C., aged thirty-eight, in November, 1884, had lumbago and neuralgia in neck from getting wet. It was treated with linaments and Morphia injections, and was followed by left sciatica. About three weeks ago had return of the neuralgia, for which she took Quinine and Iron, but without benefit. Present symptoms: The pain begins in nape like a rheumatic stiffness, worse on left side of neck; relieved by wrapping it up in woolen shawl, but worse by heat of fire; better by leaning back with head leaning on something. The pain comes on in daily paroxysms, except on 24th and 25th, during menses. The first came on in evening, but now in morning, and to-day she woke with it. The pain begins in nape, extending over occiput to vertex, and a little into ears. During the attacks, utter dislike to wine, even of the kinds which she likes. Has noticed the attacks *worse before damp weather.* Feet tender all over, worse on top and in left foot. Neck better by rubbing. Sometimes has several paroxysms of pain during day. Comes on little toes, worse on left, paining most in hot weather. *Rhus tox.*$^{101m}$ (F. C.) thrice daily for eight days.

June 5th.—Paroxysms have not come on so frequently, nor have lasted so long, till yesterday, when they were very bad. Once has been free from the attacks for two consecutive days. Last dose was taken yesterday morning. Feet less tender yesterday; felt very weak with the pain. Since taking the medicine, and not before, palms have felt subjectively damp, but were not so; also once the same sensation on vertex. Soon after commencing the medicine, had on two occasions an aching in left arm. Yesterday there was great intolerance of noise. No medicine.

June 13th.—Has had threatenings of the pain, but no real attack since. Feet much less tender. No return of dead feeling of palms, or of aching of left arm. Corns have not troubled her much.

June 22d.—No more attacks of pain even from draft; has been altogether more free from pain than the previous week. Feet rather more tender the last two days.

July 22d.—Has been, on the whole, very well, and has been able to go out to entertainments, which she could not do before without a bad attack. Has only had a few threatenings of the pain. Feet have been tender again the last two weeks, since the weather became very hot. *Rhus*$^{mm}$ (Fincke) twice daily for fourteen days.

August 1st.—Feet less tender. Has had a little of the old pains in the left hip and thigh. No medicine.

February 15th, 1887.—Consulted me for a catarrh. Has had no return of the old symptoms, except that the feet have been very tender for four or five months.

(20) *Sulphur*$^{dm}$ (F. C.) cured an obstinate and regular desire for stool, at five or six A. M., keeping her awake for an hour or so, then she is obliged to rise to get relief; it came on daily for three weeks or more.

(21) *Sepia.*—July 5th, 1888.—Mr. W., aged fifty-five, caught cold six or eight weeks ago, followed by "inflamed pain" in abdomen, relieved by hot flannels. Now has loss of appetite; *cannot always swallow solid food, but must take it out of mouth; keeps turning it over in mouth, but cannot swallow it. Driving*

*causes pain in* hypochondria, *abdomen,* and lumbar region, with desire to lie down. Full feeling up from abdomen to throat; it comes on from eleven A. M., to one P. M., and then lasts all day; better by resting. *Sepia*$^{cm}$ (F. C.) one dose.

July 21st.—Appetite better. Pains when driving altogether better. The full feeling went in three or four days, and has not returned.

September 22d.—Reports that he became quite well in two weeks, and has remained so.

(22) *Fagopyrum.*—Sept. 15th, 1888.—Mrs.——, for a week, *on waking* in morning, *pain in top of left shoulder,* rather posteriorly, extending up neck; it is a dull, bruised pain, worse on moving the part; it goes off after breakfast, but returns somewhat two or three times during day. Increased constipation for a week. For about two weeks, and worse for last week; *nausea a little before* eleven A. M., lasting thirty minutes, but without vomiting; with the nausea, and lasting afterward, has dull pain in left temple. Shoulder also feels painful if she wakes in night. For the last few days has felt hot all over, though the weather has been cooler. *Fagopyrum* $^{1m}$ (F. C.), one dose.

September 21st.—On 16th the pain in shoulder on waking was much worse; much better by night. On 17th, less pain on waking, and none since till this morning, when she had a little on waking. No more constipation. Very little return of nausea, and no pain in temple. Still feels hot, but less. No medicine.

October 3d.—Reports that none of the symptoms had returned. This belongs to an interesting and complicated case of tumor, which I hope, when completely cured, to publish in full. In the meantime, I give this extract to illustrate the action of a comparatively new remedy.

Here is another verification:

Miss L., aged thirty-four, had chronic diarrhœa. On April 25th, 1888, she reported that it had increased; generally *commencing about six or seven* A. M.; also *sinking in stomach,* as if she wanted food, coming on suddenly *about six* P. M. *Fagopyrum*$^{1m}$ (F. C.), one dose.

The sinking was much worse for about a week after the dose, then improved, and almost entirely ceased. The diarrhœa improved for some time, but afterward increased, and I had to prescribe *Lachesis.*

Symptoms 336, 426, 358, 602–6, 612–14, in *Encyclopædia* are thus verified: *Fagopyrum* has over eight hundred symptoms, but I have seen no cures reported by us.

(23) In a case of chronic metrorrhragia, occurring in a woman sixty-eight years old, and with weak heart, the following symptoms were removed. Each remedy I gave in this case acted promptly, and I hoped to cure her, but, after great improvement, the weak heart suddenly failed, and she died in a few hours. This is the only death that has occurred in my practice since May, 1885, and that case was a death at age of seventy-six from old age. Single doses of the following remedies were given:

*Thuja*$^{cm}$ (F. C.) cured dislike to fresh meats and to potatoes.

*Terebinthina*$^{cm}$ (Fincke) cured: warm drinks cause, in their descent, pain in sternal region, middle part, with tenderness to touch; the pain is somewhat burning.

*Colchicum*$^{cm}$ (F. C.) cured constipation, with constant, ineffectual desire, but only passes a little clear, transparent, colorless jelly with some froth; the urging to start is accompanied with sharp pain in rectum and bowels; better after the jelly has passed.

*Sanicula*$^{cm}$ (F. C.) cured dirty-brown discharge from uterus, with horribly putrid odor, like a battle-field after a few hot days; the discharge came in hot gushes.

*Carbolic acid*$^{45m}$ (Fincke) cured an aversion to tea, of which she was usually fond; and much improved a diarrhœa.

*Sulphur*$^{dm}$ (F. C.) cured uterine discharge, watery, whitish, copious, in gushes and scalding; sometimes thicker, whitish-yellow, staining the napkins a mustard-yellow, with yellow granules like crushed mustard seed, and fecal odor.

(24) *Phosphorus.*—April 18th, 1887.—Miss W., aged sixty-three, has had *much brain work.* Constant fidgets since Christmas; occur two or three times daily, *always at seven* P. M. whether she dines early or late. The attack begins either with sleepiness, or with *pricking* or irritation in *various spots on skin,* as if something had bitten her; then a contractive feeling in one or other thigh, as if the parts were drawn together, *compelling her to walk,* or stand, or use the leg, which temporarily relieves it; if she does not move the leg, it causes pain in sacral region and occiput. At seven P. M., she feels as if the day was over, and that she ought to go to bed. Sleep, sometimes long and heavy, sometimes only from two A. M. to five A. M.; has lain awake till six A. M. With the contractive feeling, sometimes has palpitation. Often has shuddering, and cutis anserina, with the fidgets. Occasionally had these fidgets when young. A brother has them if fatigued.

*Phosphorus*$^{cm}$ (F. C.) twice daily for one week, cured her speedily.

